# QUaternary fault strain INdicators database - QUIN 1.0 - first release from the Apennines of central Italy

**DOI:** 10.1038/s41597-022-01311-8

**Published:** 2022-05-12

**Authors:** Giusy Lavecchia, Simone Bello, Carlo Andrenacci, Daniele Cirillo, Federica Ferrarini, Noemi Vicentini, Rita de Nardis, Gerald Roberts, Francesco Brozzetti

**Affiliations:** 1 DiSPuTer, Università degli Studi “G. d’Annunzio” Chieti-Pescara, Chieti, Italy; 2 CRUST - Centro InteRUniversitario per l’analisi Sismotettonica Tridimensionale, Chieti, Italy; 3 Department of Earth and Planetary Sciences, Birkbeck, University of London, London, UK

**Keywords:** Tectonics, Structural geology

## Abstract

We present QUIN, a “QUaternary fault strain INdicators database”, designed to integrate and unify published and unpublished local-scale geological information and derive strain parameters for structural and seismotectonic analyses. It provides data on 3339 Fault Striation Pairs (FSP; fault plane and slickenline), distributed within 455 survey sites. These are exposed along the intra-Apennine Quaternary extensional faults of Central Italy. The area covers an extent of ~550 km in a NW-SE direction. We give information on FSP location, attitude and kinematics, and deformation axes. We also provide an original shapefile of the faults hosting the FSP. A large amount of homogeneously distributed Quaternary fault/slip data help to clarify and implement the contemporary geometric and kinematic deformation pattern of Central Italy that appears scattered and incomplete whenever exclusively derived from earthquake data. The high-density of structural data can help investigate stress pattern heterogeneities at local scales, with relevance for new generations of hazard assessment evaluation and a better understanding of rupture propagation and related barriers.

## Abstract (summary)

**Table:** 

Measurement(s)	active geological fault
Technology Type(s)	geological tools
Sample Characteristic - Environment	Quaternary faults • Apennines
Sample Characteristic - Location	Italy • Northern Apennines • Central Apennines • Tuscan-Emilian Apennines • apennines

## Background & Summary

Open-access databases of present-day stress patterns, at regional to local scale (>500 km to <100 km), are available for most of the world^1^ and sometimes for specific regions, such as Italy^2^. The stress pattern is defined by indicators such as focal mechanisms, borehole breakouts, overcoring data, and active fault geometries, and it is generally represented using horizontal stress orientations. Geological fault data, which can elucidate local stress variations^3^, relevant at the seismotectonic scale, are generally subordinate. The World Stress Map (WSM) contains ∼43,000 records, of which ~75% are derived from earthquake focal mechanisms, ~22% are derived from wellbore breakouts, drilling-induced fractures, and *in-situ* stress measurements (overcoring, hydraulic fracturing, borehole slotter), while only ~3.1% are derived from fault-slip data. The last release of the Italian crustal stress database (IPSI 1.4)^2^ contains 928 records, of which ~2% are derived from geological-structural data. The information is given in the form of horizontal maximum and minimum stress, but the original data on the fault/slip population are not available.

The global under-utilization of geological data in active stress databases may be partly attributed to the limited number of well-exposed faults recognized as Late Quaternary (last 0.126my) in age and to a lack of systematic collaboration amongst the geological community. Although fault/slip data have been available in the literature for the last forty years, they are in general not systematically organized and easily usable. We consider that in areas such as Italy, where, according to several authors, although not all^4–6^, the regional stress field has remained unchanged over the last few million years, the analysis of structural data relevant for seismogenic purposes can be extended at least to the overall Quaternary time interval (last 2.58 my)^7^. Furthermore, this assumption makes long-term surface fault-slip data valuable to better and more extensively constrain the present deformation pattern and the corresponding crustal state of stress. The active crustal stress pattern is likely to be less well represented if exclusively derived from instrumental earthquake data.

Recently, structural-geological databases have started to become available^8–13^. Faure Walker *et al*.^12^ presents an extensive fault-slip database of 166 sites with complete dip, dip angle, plunge, and azimuth data, to gather well-constrained late Quaternary geological data for seismic hazard assessment in Central Italy. A local database of the coseismic surface effects following the 2016 Norcia earthquake (M_w_ 6.5) is made available by Villani *et al*.^8^, which provides ~7000 records surveyed along the Mt. Vettore fault, of which 450 give complete information on dip/dip-direction, plunge/azimuth, and the rake for 97 data collected over an along-strike extent of a few tens of kilometres.

**Fig. 1:**
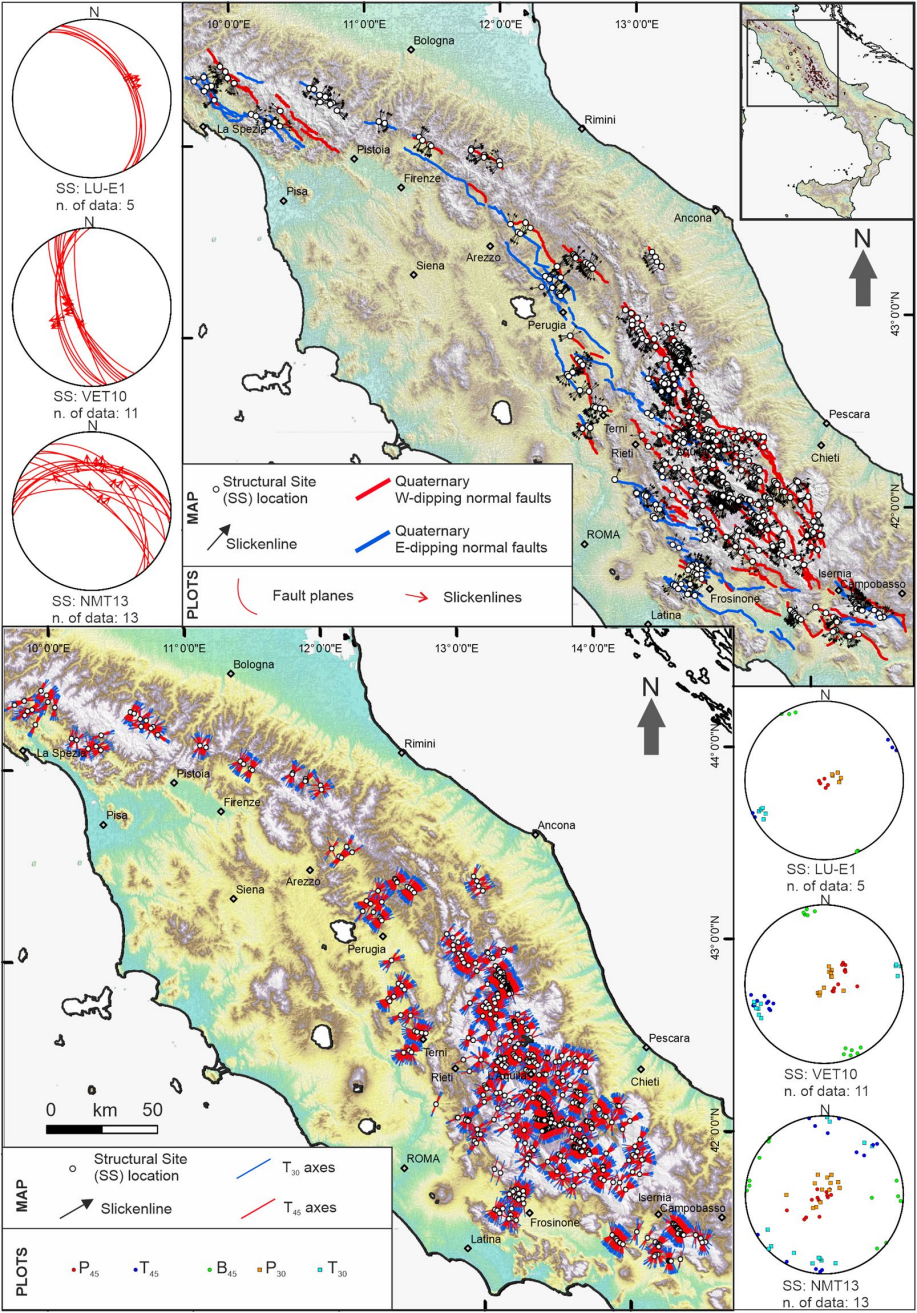
Map view of the Host Faults and the QUIN database. (**a**) Structural Sites (SS) and slip vectors from the Fault/Striation Pairs (FSPs) data in the QUIN database; the SS are projected on a shaded relief map of northern-central Apennines of Italy along the trace of Quaternary normal faults hosting the SS; fault traces are derived from the Host Fault database (see next section). Stereoplots (Lambert equal-area, lower hemisphere projection) at the map side represent examples of FSP data for three SS chosen across the Quaternary fault belt; key: LU-E1, VET10, NMT13 = SS short names as reported in the QUIN database. (**b**) T_30_- and T_45_-axis computed in this database, on a shaded relief map of northern-central Apennines of Italy. Stereoplots on the right of the figure represent the same SS as in panel **a** (same location), with the axes of the deformation obtained with the methodology described in the main text. Key: P_45_ and P_30_ = contractional axes, B_45_ = neutral axes; T_45_ and T_30_ = extensional axes.

The main contribution of this paper (*i.e*., the first release of the QUIN database) is that it contains 3339 Fault-Striation Pair (FSP) records, consisting of both newly acquired data (1315) and data from the literature (2024) distributed within 455 structural sites (SS) located along the major Quaternary intra-Apennine faults of central Italy (Fig. 1a). The unpublished data are recovered from our field notebooks and maps collated over the last few decades, augmented with new fieldwork to fill information gaps. We focus on ~550 km along-strike of the Quaternary extensional belt of central Italy, for a total area of about 30,000 km^2^. The belt consists of interconnected normal and normal-oblique faults, responsible for earthquakes with M_w_ up to 7.0^14^ in a densely inhabited territory.

For each FSP, we provide information on the fault hosting the SS (hereinafter referred to as “Host Faults”), the geometric-kinematic parameters, and the quality parameters. Furthermore, we calculate the attitudes of the corresponding principal deformation axes, considering two scenarios and assuming the contractional axis at 45° and 30° in the slip plane (Fig. 1b). This choice is based on the observation that, at upper-crust levels, Andersonian rules assume that the rupture plane is orientated at 30° with the principal stress (σ1)^15^. At the same time, classic P-T deformation axes, measured at 45° and derived from focal mechanisms, may be compared for a preliminary representation of the SH_min_ horizontal trajectories^16^.

FSP from our QUIN database contains areas that have undergone recent earthquake activity, but also contains areas that have been aseismic in instrumental times, which may need special attention for forthcoming seismic hazard analysis. The QUIN database contains comprehensive and homogenous information on Quaternary fault/slip and the related deformation field to be implemented with earthquake data to perform formal stress inversions at regional scales and investigates local-scale effects controlling active deformation, seismogenic faulting, and seismic hazard, in a region of high-seismogenic potential. In addition, it may be useful for further elaborations on structural analysis, geodynamic interpretation, geothermal and petroleum exploration.

## Methods

### Host faults

Well-exposed normal and normal/oblique fault systems with activity since Early Quaternary times (last 2.58 My^17^) and, in some cases, Late Pliocene, are distributed across the mountain belt of peninsular Italy^18^. They crop out in different lithotypes, including platform-to-basinal Meso-Cenozoic limestones, Cenozoic siliciclastic turbidites, and Plio-Quaternary clastic infill of continental basins.

An original database of the fault traces hosting the SS is stored in ZENODO^19^. The Host Fault database functions to precisely locate each SS on a specific fault segment. Fault traces available in a pre-existing database (Fault2SHA^12^), which covers the central-eastern sector of our study area, were not used for two main reasons: 1) need of homogeneity with the rest of the area not covered by Fault2SHA; 2) a better geographic fit between the SS and our fault traces, that were partially surveyed in the years during the structural data mapping, although spatial misfits between the two datasets are generally very small (<a few kilometres).

The Host Fault attribute table provides information on fault location, name and dip-direction, and the age of activity of the fault system containing the Host Faults. The fault traces were derived from the literature and locally from unpublished maps related to the newly acquired FSPs. We started from the online version of the Structural and Neotectonic Models of Italy (scale 1:500,000, CNR^20,21^) and updated it with more detailed traces of Late Pleistocene-Holocene faults available within online maps and repositories^12,22–25^. We also used some raster maps available online, among which the sheets of the Geological Map of Italy at scale 1:100,000 and 1:50,000, and the seismotectonic map of the Emilia-Romagna Region at scale 1:250,000^23^. Detailed information used to draw the fault traces was derived from all the papers used to compile the QUIN fault/slip database (see following sections).

The age of the fault systems and the evidence of earthquake activity in historical and/or instrumental times were derived from the literature^4–6,12,14,26–31^ and unpublished biostratigraphic data.

The age of onset of the fault system extends across the time interval from Late Pliocene to Middle Pleistocene and, at a regional scale, is progressively younger towards the east^4–6,26–29^. All the Host Faults may be considered potentially seismogenic and of interest for seismic hazard assessment evaluation, either because of direct geological and/or seismological evidence of activity in Late Pleistocene-Holocene times or because they belong to Late Pliocene-Quaternary fault systems nucleated and developed during the present seismotectonic regime. Details on the age of the oldest and the youngest evidence of the fault activity may be found within the attribute table of the Host Fault shapefile provided with this paper^19^. Overall, all the Host Faults can be considered undifferentiated Quaternary in age.

In map view, the Host Faults delineate a NW-SE to NNW-SSE striking broad Quaternary tectonic province consisting of west- and east-dipping normal and normal-oblique faults (Fig. 1a). The west-dipping normal faults, which in places have controlled the growth of Late Pliocene-to-Quaternary basins, show evidence of Late Pleistocene to present activity^12,32^. Although characterized by a less evident morphological expression, the east-dipping normal faults show also show geological and seismological evidence of recent activity^6,33–36^. Indeed, earthquake data (*i.e*., hypocenters and focal mechanisms) and seismic lines have led many authors to suggest that the west- and the east-dipping outcropping Quaternary faults detach at depth onto a common east-deepening basal detachment. This geometry is largely accepted in northern Italy^37–42^, but only recently suggested in central-southern Italy^36,43–45^.

### Steps for building the QUIN database

To build a unified and comprehensive database, we followed the following sequential steps:

1. We recovered published FSP data (only data with both fault plane and slickenline attitudes) surveyed along the major intra-Apennine faults of Central Italy. Where the fault-slip attitudes were not tabulated in the original data source, we graphically derived them from their stereographic representation within the original paper, using the Stereonet software^46,47^. We applied corrections to the projection of slickenlines attitudes on the fault plane (see next section).
2. We extracted unpublished data from our field booklets and accompanying maps for FSP data surveyed in the last few decades. We projected the corresponding SS in map view on a GIS platform and identified information gaps.
3. We performed new field campaigns (from 2018 to 2021) to fill gaps in data and locally integrate the information.
4. With the previous steps, we recovered a total of 3339 FSP (2024 from literature and 1315 original), measured at 455 survey sites (SS). Each FSP corresponds to the raw data record of the QUIN fault/slip database.
5. We identified with a quality code the precision in reading the FSP attitudes from the original stereoplots (see the Data Records section).
6. We identified the spatial coordinates of each SS by georeferencing the location maps from the literature or our booklets. In some cases, Latitude and Longitude coordinates were available. The precision of the geographic location was also identified with a quality code (see the Data Records section).
7. Starting from the FSP data, we calculated the rake values (Aki and Richards format) and classified the FSP in kinematic classes corresponding to pitch ranges of 0–30° for strike-slip faults, 30–60° for oblique faults, and 60–90° for dip-slip faults (see the Data Record section).
8. We calculated the principal deformation axes from the FSP data and/or from the plane attitude and rake (see the section “Calculation of Deformation axes”), using a combination of MATLAB algorithms^48^ for fault-slip kinematic analysis developed for this paper.

### Fault/striation pairs and structural sites

The FSPs in the QUIN database refer to meso- and major-scale fault planes (see numbered list for details) surveyed in Structural Sites (SS) located along or near to the Host Fault traces. We did not report fault planes without lineation data as they are unsuitable for kinematic analysis.

The SS are separated into four groups, based on their structural features and the position with respect to the Host Faults:

1. SS located along the Host Faults, just on the main fault plane or near it, within the related damage zone (<500 m). In the former (on-fault SS), the FSP data represent both the major fault plane and the associated mesoscopic splays; in the latter (near-fault SS), the FSPs represent the mesoscopic fault planes detected in proximity to the major tectonic contact. The on-fault and near-fault structures are primarily collected in the sectors of the Apennines where normal faults displace, at the surface, carbonate sedimentary successions. These faults are often characterized by spectacular and continuous exposures of fault planes and by highly developed damage zones. For these reasons, they are the most common and frequently detected structures and, therefore, the most numerically present in our database (88.2%) (Fig. 2). The near-fault SS are also observed in sectors where well-stratified, calcareous or siliciclastic turbidite succession crop out^49^.

**Fig. 2:**
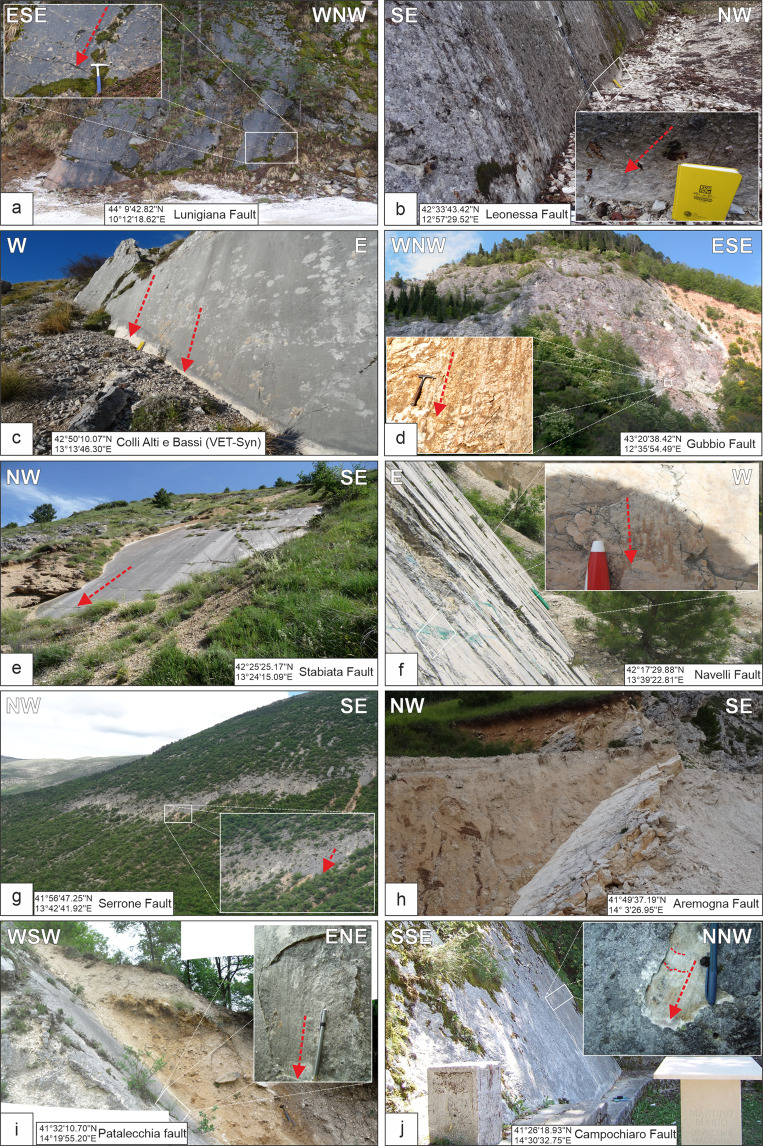
Examples of striated fault planes from the Apennine extensional belt showing the characteristic field aspect of the most common QUIN’s FSPs. The location of the outcrops is reported as coordinates in each panel; the fault striations are represented as red arrows.
2. SS of syn-kinematic synthetic and antithetic mesoscopic faults. These are surveyed outside the Host Fault damage zone, most commonly within the hanging wall blocks, at a distance from the major fault trace generally >500 m. These types of SS appear to be particularly widespread within the continental deposits filling the Quaternary syn-tectonic basins. These are ~6.5% of the total.
3. SS of syn-kinematic major and/or mesoscopic faults surveyed along the trace of antithetic structures to the Host Fault. These are ~2.9% of the total.
4. SS of scattered Quaternary mesoscopic fault planes with strike transversal to the major fault alignment (~2.4%).

We report the above classification in our database together with the other information described in the manuscript.

### Combination of MATLAB scripts for fault-slip kinematic analysis

**Fig. 3:**
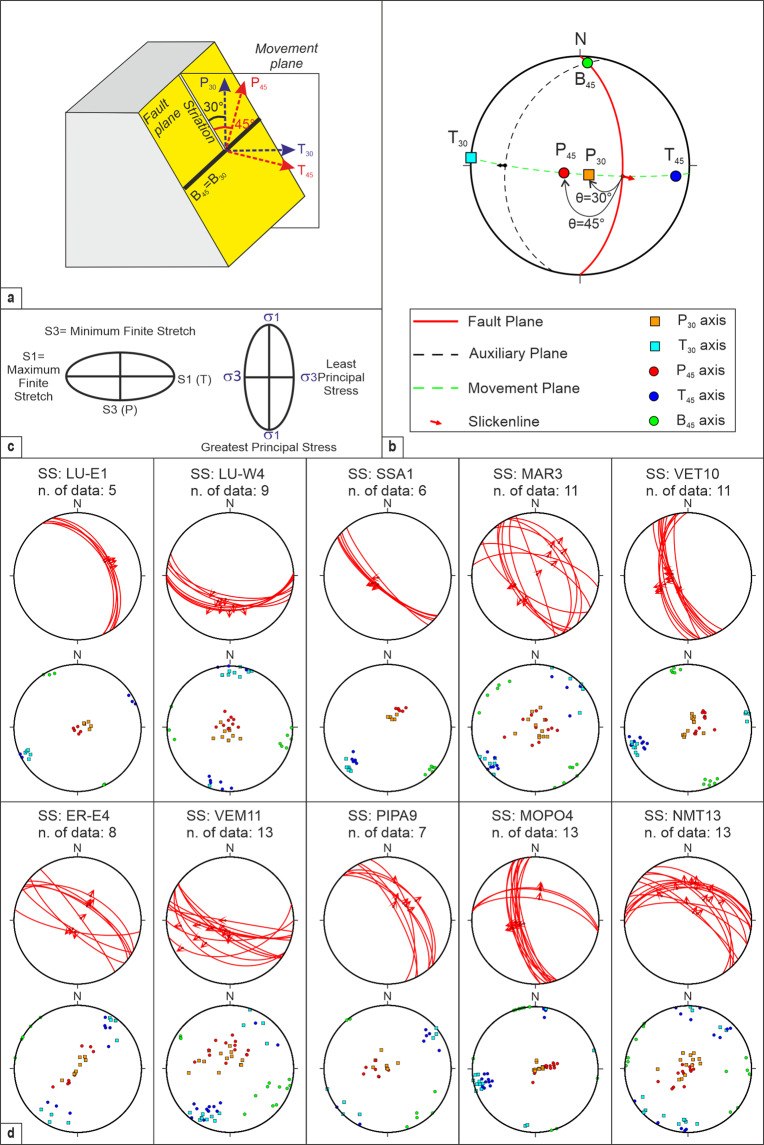
FSP structural analysis performed using the combination of MATLAB scripts developed for this paper^48^. (**a**) Schematic representation of the fault geometry used to calculate the P_45_- B_45_- T_45_ and the P_30_- B_30_- T_30_ deformation axes; the block diagram is modified from Célérier^15^. Key: M, plane of movement^54^. P_45_ and P_30_ = contractional axes, B_45_ and B_30_ = neutral axes; T_45_ and T_30_ = extensional axes. (**b**) Example of stereographic representation of the geometrical relationships among fault plane, slip, directions, deformation axes, angle θ computed for each FSP of this database (modified from Sippel *et al*.^16^). (**c**) Strain ellipse configuration; for an ideal Andersonian condition, the principal strain axes (S1, S2 and S3, where S1 ≥ S2 ≥ S3) are coaxial to the principal stress axes (σ1, σ2 and σ3, where σ1 ≥  σ2 ≥  σ3). The long axis of the finite strain ellipse is the direction of maximum finite stretch (S1); the short axis is the direction of minimum finite stretch (S3); *vice versa* for the stress ellipsoid. (**d**) Examples of FSP data stereographic projection (equal-area net, lower hemisphere) using the graphical representation adapted in Andrenacci *et al*.^48^; each stereoplot refers to a specific SS identified in the QUIN database; Key: LU-E1, LU-W4, SSA1, MAR3, VET10, ER-E4, VEM11, PIPA9, MOPO4, NMT13 are the SS short names as in the QUIN database. Legend as in panel b.
- Graphical representation of planar (fault plane) and linear (striation and deformation axes) data in a lower hemisphere Schmidt net (see examples in Fig. 3d).

To perform kinematic analysis of fault-slip data, we prepared a new compilation of simple graphical and numerical algorithms in MATLAB^48^ valid for:

- Calculation of rake from fault and slip vector attitude and sense of motion.
- Calculation of slip vector (trend and plunge) from fault attitude and rake.
- Conversion of rake into pitch.
- Calculation of the conjugate dihedral plane with its slip vector.
- Calculation of deformation axes, assuming variable rupture angles θ between the slip vector and the least extensional axes (P_45_ or P_30_ in Fig. 3a,b).

Equivalent calculations and graphical representations are incorporated in other well-known software, such as Faultkin^50,51^, TectonicsFP 1.6.5 (https://github.com/freiter/TectonicsFP.git) (*e.g*.^52^,), but, with our in-sequence combination of algorithms, it is possible to obtain all the geometrical-kinematic information (described below) on the FSP in just one export. This makes our code a valuable and easy-to-use tool for fault analysis and representation, usable for seismotectonic and structural geology purposes. A complete guide to using our code is available in the ZENODO repository^48^.

### Calculation of deformation axes

The deformation axes, generally referred to as P (contraction), B (neutral) and T (extension), as well as the stress axes, referred to as σ_1_ (compression), σ_2_ (intermediate) and σ_3_ (tension), are the direct representation of fault attitude and sense of slip^15,53^. Both P and T and σ_1_ and σ_3_ lie on the movement plane^54^, perpendicular to the intermediate or neutral axis, the slip direction being parallel to the maximum resolved shear stress^55^. If the state of stress associated with active faults near the surface is compatible with Anderson’s theory, we expect a principal direction to be near-vertical with the other two being near horizontal and perpendicular to each other. Also, an approximation of B, T and P-axes of focal mechanisms with stress-axes may be justified if an angular error of ~15° is accepted between P and σ_1_^56^ (Fig. 3). There may be significant differences between the principal stress and strain axes in areas with pre-existing fractures. The greatest principal stress (σ_1_) may occur virtually anywhere within the P-quadrant, and the least principal stress (σ_3_) likewise anywhere within the T-quadrant^57^. Therefore, the association of a stress field to a given complex fault population needs formal inversion of fault-slip data. Some authors (*e.g*.^58^) observe that relevant strain variations occur with proximity to fault tips and that, therefore, only slip-directions from the central portion of the fault are meaningful to constraint regionally significant stress patterns.

Our intent with the QUIN database is to compute, with a simple geometric approach, the likely principal deformation axes of any FSP, given that any striated fault, even if mesoscopic in size, may help to place local geometrical constraints on them^59^.

To compute the three principal deformation axes of each FSP datum, we follow the kinematic method based on the Mohr-Coulomb fracture criterion, known as PBT-Method^16,60^. Data inputs are the attitude of the fault plane and its striation with sense of slip, plus an *a priori* defined fracture angle θ between the shortening axis and the slip vector on the fault plane. Generally, a constant value of the angle θ for all faults of a given fault system is assumed without considering the anisotropy of the involved materials^16^.

The angle θ between the fault plane and the compressional axis may vary from 0 to 90°. However, in the literature, it is generally assumed to be equal to 45° when considering the dihedral configuration between two complementary planes^53^, as is typical for focal mechanisms, or as 30° when considering the Mohr-Coulomb criterion and the Anderson model^15,61^.

Sippel *et al*.^16^, among others^61,62^, highlight that an angle of 30° is appropriate for natural fault-slip data at upper crustal levels, as well as for brittle deformation in the laboratory^63–65^. A value of 30° is assumed in the World Stress Map for paleostress reconstruction from fault/slip data measuring the P-axis at 30° to fault in plane of slip vector^66^.

QUIN 1.0 gives an estimation of deformation axes directions calculated for each FSP, assuming both θ = 30° and = 45°, as two alternative possible kinematic scenarios. The axes calculated with θ = 45° are referred to as P_45_ (contraction or shortening), B_45_ (neutral) and T_45_ (extension or stretching); those calculated with θ = 30° are referred to as P_30_, B_30,_ and T_30_ (Fig. 3a and b). In both cases, the fault plane contains the intermediate axis (B) and the fault striation with its slip direction and is perpendicular to the movement plane (Fig. 3a). Therefore, an approximation of the P-B-T axes with the principal stress axes (σ_1_ ≥  σ_2_ ≥  σ_3_) may be assumed in Andersonian conditions typical at upper crustal depths^15^.

To compute the attitude of the two types of deformation axes, we adopted a simple geometric procedure described in Andrenacci *et al*.^48^ (Fig. 3). We first calculated the attitude of the neutral axis (B) located on the fault plane at an angle of 90° from the slip vector. Then, on the Movement plane, we measured an angle of 30° and 45° toward the vertical to obtain P_30_ and P_45_, respectively. Finally, at 90° from P, on the movement plane, we measured T_30_ and T_45_, respectively.

## Data Records

### QUIN 1.0 database

QUIN 1.0 provides a comprehensive compilation, from literature and new work, of fault/slip data for intra-Apennine Quaternary faults at well-distinguished SS, and new computations of corresponding kinematic parameters (rake and deformation axes).

QUIN 1.0 is stored in the Pangaea repository as shapefile (.shp) and text (.txt) format^67^. It contains 3339 unified FSP records, 2024 extracted from the literature and further elaborated, and 1315 new data presented and elaborated in the present data descriptor.

The records are organized into 34 fields. Each record refers to a FSP, surveyed on a major Quaternary fault plane or close to its trace. Each FSP is part of a SS containing more than one FSPs. The 3339 FSP are distributed within 455 SS.

The 34 fields of the database are subdivided into three thematic groups (A, B, C) defined as follows:

A) FSP identification and SS location (fields 1 to 12).
B) FSP geometry, quality ranking and references (fields 13 to 22).
C) FSP deformation axes (fields 23 to 34).

Below we describe the individual records belonging to the three groups.

### FSP identification and SS location

1. Ordinal Number (short name: Ord_No): uniquely identifies each Fault Striation Pair (FSP).
2. Survey Site (short name: SS): contains one or more FSPs.
3. FSP identification Code (short name: FSP): is expressed as the acronym of the fault containing the Survey Site, plus a cardinal number which refers to the Survey Site (SS) and a lowercase letter to identify the individual FSP.
4. Longitude (short name: Lon) of the SS in decimal degrees (dd.mmmmm), in the WGS84 reference frame.
5. Latitude (short name: Lat) of the SS in decimal degrees (dd.mmmmm), in the WGS84 reference frame.
6. Region (short name: Reg): administrative region within the boundaries of which the data is located.
7. Locality (short name: Loc): municipality within the boundaries of which the data is located.
8. Position (short name: SS_Pos): position and prevailing dip-direction of the SS with respect to the Host Fault: (1 = the SS is on-fault, located on the Host Fault trace, or near-fault, at a distance less than ~500 m and the FSP’s prevailing dip-direction is synthetic with the Host Fault; 2 = the SS is off-fault, e.g. at a distance >~500 m from the Host Fault trace and/or within the hanging-wall syntectonic basinal deposits; 3 = the SS is on a subsidiary antithetic fault and is characterized by prevailing opposite dip-direction to the Host Fault; 4 = the SS prevailing direction is transversal to the Host Fault main trend).
9. Coseismic Displacement (short name: Seism); it reports if the SS is located on a fault plane displaced during a recent (*i.e*., instrumentally recorded) seismic event and the FSP measurement refer to a coseismic discontinuity (the fault acronym and date of the associated earthquake is reported, *e.g*. AQ_2009).
10. Host Fault (short name: Fault): name of the individual Quaternary fault hosting the SS.
11. Host Fault Acronym (short name: F_Acronym): acronym of the fault system hosting the SS.
12. Host Fault Dip-Direction (short name: F_DipDir): approximate direction of the major fault, and/or fault system dip to which the SS and its FSP belong, with respect to the North. An example of this data group is given in Table 1.

**Table 1:** FSP identification and SS location; example of 10 records.

Ord_No	SS	FSP	Lon	Lat	Reg	Loc	SS_Pos	Seism	Fault	F_Acronym	F_DipDir
984	VENT1	VENT1c	13.17633	42.78252	Umbria	Norcia	1		Mt_Ventosola	VENT	WSW
985	VENT1	VENT1d	13.17633	42.78252	Umbria	Norcia	1		Mt_Ventosola	VENT	WSW
986	VET14	VET14e	13.26124	42.78218	The_Marches	Arquata_del_Tronto	2	Central_Italy_2016	Vettoretto	VET	W
987	MTB-E1	MTB-E1j	12.40448	42.78162	Umbria	Todi	2		Middle_Tiber_Basin_E_dip	MTB-E	NE
988	MTB-E1	MTB-E1k	12.40447	42.78162	Umbria	Todi	2		Middle_Tiber_Basin_E_dip	MTB-E	NE
989	MTB-E1	MTB-E1l	12.40447	42.78162	Umbria	Todi	2		Middle_Tiber_Basin_E_dip	MTB-E	NE
990	MTB-E1	MTB-E1m	12.40447	42.78162	Umbria	Todi	2		Middle_Tiber_Basin_E_dip	MTB-E	NE
991	MTB-E1	MTB-E1n	12.40447	42.78161	Umbria	Todi	2		Middle_Tiber_Basin_E_dip	MTB-E	NE
992	MTB-E1	MTB-E1o	12.40447	42.78161	Umbria	Todi	2		Middle_Tiber_Basin_E_dip	MTB-E	NE
993	MTB-E1	MTB-E1p	12.40447	42.78161	Umbria	Todi	2		Middle_Tiber_Basin_E_dip	MTB-E	NE

### FSP geometry, quality ranking and references

13) Strike (short name: Strike – expressed in degrees): azimuth angle of a FSP plane with respect to the North.
14) Dip direction (short name: Dip_dir – expressed in degrees): direction of the FSP plane dip with respect to the North, expressed with the right-hand rule strike direction (Table 2).
15) Dip angle (short name: Dip – expressed in degrees): angle of dip of the FSP plane measured.
16) Trend (expressed in degrees): direction of the FSP slip vector.
17) Plunge (expressed in degrees): dip of the FSP slip vector.
18) Rake: calculated using the Aki-Richards annotation (normal and reverse faults yield rake angles of −90° and +90°, respectively, whereas strike-slip faults yield rake angles of 0° and ±180°).
19) Rake-based kinematics (short name: Kin): provides information on the fault regimes classification (PN = Pure dip-slip Normal fault; NF = Normal fault; NS = Normal Strike; SN = Strike Normal; SSL = Strike-Slip Left; SSR = Strike-Slip Right; PSS = Pure Strike-Slip). Further information on rake ranges can be found in the guide^48^.
20) Location quality ranking (short name: Q_loc): this expresses with three quality classes (A, B, C) the precision in the geographic location of each SS and its position with respect to the trace of the Host Fault: A = precise location from available GPS data and/or available tabulated data sets or from original field map (expected error in the order of few meters to some tenths of m); B = approximate location from clear maps and sketch (expected error in the order of some hundreds of m); C = approximate location from not fully clear maps and sketch (expected error up to 1 km).
21) Data resolution quality ranking (short name: Q_res): this expresses with three quality classes (A, B, C) the precision in reporting the FSP attitude data (fault plane and its striation) from the original reference source to the QUIN database: A = precise data from tablets and published tabulated data set or original field booklets; B = FSP attitudes derived from clear and accurate published stereoplots projections (expected reproduction error in the order of 1–2°); C = FSP attitudes derived either from not so clear and easily readable published stereoplots projections or from fixed −90° rake for dip-slip faults (expected error up to about 5°, described in Technical Validation section).
22) Reference (short name: Ref): source paper used for deriving the Survey Sites location (Lat and Lon) and the FSP attitude (strike, dip, dip-angle, trend, and plunge). All remaining data from this database are calculated in this work. Data reference key: Ada12^68^; B&L94^69^; B&L95^70^; Bar91^71^; Bon00^72^; Bon16^73^; Bon95^74^; Bon96^75^; Boni13^76^; Boni16^32^; Bro19^77^; Cal92^78^; Cal95^79^; Cal99^80^; Col03^81^; DAg98^82^; DB05^83^; DB11^84^; DD14^85^; Fer15^86^; Fer17^87^; Fon20^88^; FW10^89^; FW12^90^; FW19^91^; G&G20^92^; Lav12^93^; Lav17^36^; Mil19^94^; Mira11^95^; P&P04^96^; Pace02^97^; Pap05^98^; Per18^99^; Pic99^100^; Piz10^101^; R&M04^102^; Vig20^29^; Vil18^8^; Wil15^103^. Unpublished data from this paper are labelled as “PP” (*i.e*., present paper). An example of this data group is given in Table 2.

**Table 2:** FSP geometry with quality ranking and references; example of 10 records.

Strike [deg]	Dip_dir [deg]	Dip [deg]	Trend [deg]	Plunge [deg]	Rake [RHR]	Kinematics	Q_loc	Q_res	Ref
89	179	49	196	48	−101.3	PN	B	B	Boni16
90	180	46	158	44	−74.4	NF	B	B	Boni16
88	178	45	212	40	−115.4	NF	B	B	Boni16
87	177	45	181	45	−92.8	PN	B	B	Boni16
89	179	39	251	14	−157.3	SS	B	A	PP
34	124	55	59	31	−39.0	SN	B	A	PP
10	100	45	113	44	−99.3	PN	B	A	PP
334	64	39	58	39	−85.3	PN	B	A	PP
331	61	54	21	47	−64.0	NF	B	A	PP
321	51	40	40	40	−81.6	PN	B	A	PP

### FSP deformation axes

23) Trend of P_45_-axis (short name: P45_trend – expressed in degrees): trend of the shortening axis.
24) Plunge of P_45_-axis (short name: P45_plunge – expressed in degrees): plunge of the shortening axis.
25) Trend of B_45_-axis (short name: B45_trend – expressed in degrees): trend of the neutral axis.
26) Plunge of B_45_-axis (short name: B45_plunge – expressed in degrees): plunge of the neutral axis.
27) Trend of T_45_-axis (short name: T45_trend – expressed in degrees): trend of the extension axis
28) Plunge of T_45_-axis (short name: T45_plunge – expressed in degrees): plunge of the extension axis.
29) Trend of P_30_-axis (short name: P30_trend – expressed in degrees): trend of the minimum extension axis.
30) Plunge of P_30_- axis (short name: P30_plunge – expressed in degrees): plunge of the minimum extension axis.
31) Trend of B_30_-axis (short name: B30_trend – expressed in degrees): trend of the neutral axis.
32) Plunge of B_30_-axis (short name: B30_plunge – expressed in degrees): plunge of the neutral axis.
33) Trend of T_30_-axis (short name: T30_trend – expressed in degrees): trend of the maximum extension axis.
34) Plunge of T_30_-axis (short name: T30_plunge – expressed in degrees): plunge of the maximum extension axis. An example of this data group is given in Table 3.

**Table 3:** FSP deformation axes; example of 10 records.

P45_Trend	P45_Plunge	B45_Trend	B45_Plunge	T45_Trend	T45_Plunge	P30_Trend	P30_Plunge	B30_Trend	B30_Plunge	T30_Trend	T30_Plunge
329.5	35.7	116.4	49.4	227.0	16.8	311.2	39.7	116.4	49.4	215.0	7.4
336.2	45.6	107.2	32.8	215.8	26.4	316.5	53.6	107.2	33.8	206.5	14.1
333.1	41.9	104.7	36.5	216.4	26.5	314.5	49.6	105.7	36.5	206.2	15.0
327.0	48.6	100.7	31.3	206.5	24.1	305.6	56.2	101.7	31.3	197.9	11.5
322.5	47.7	100.4	34.0	206.2	22.0	300.9	54.3	100.4	34.0	197.1	9.8
126.8	50.7	351.4	30.2	247.3	22.6	149.6	57.9	351.4	30.2	255.6	9.8
63.5	76.0	155.3	0.4	245.4	14.0	269.1	88.9	155	0	65.3	1.0
54.9	55.7	151.9	4.8	245.1	33.8	48.3	70.5	152	5	243.6	18.9
26.8	39.7	154.4	36.3	268.9	29.4	9.4	48.1	154	36	258.3	18.0
58.6	66.0	327.9	0.3	237.7	24.0	60.0	81.0	328	0	237.8	9.0

### Host Fault database

We provide a new database of the Host Faults containing the investigated SS. The database is stored in ZENODO^19^ as ESRI shapefile (.shp). It contains 221 fault traces records and the related table contain fields providing the following information:

1. Ordinal Number (short name: Ord_No): it uniquely identifies each fault trace (from north to south).
2. Fault longitude start point (short name: X_start): longitude of the trace start point in decimal degrees (dd.mmmmm) and WGS84 reference frame.
3. Fault latitude start point (short name: Y_start): latitude of the trace start point in decimal degrees (dd.mmmmm) and WGS84 reference frame.
4. Fault longitude end point (short name: X_end): longitude of the trace end point in decimal degrees (dd.mmmmm) and WGS84 reference frame.
5. Fault latitude end point (short name: Y_end): latitude of the trace end point in decimal degrees (dd.mmmmm) and WGS84 reference frame.
6. Region (short name: Reg): Italian administrative region within the boundaries of which the data is located (In cases where a fault crosses multiple regions, these have been reported from north to south).
7. Host Fault Name (short name: H_F): name of the fault hosting the QUIN 1.0 Structural Sites or neighboring to them.
8. Host Fault Key (HF_key): gives information on the generic eastward or westward dip-direction; the additional term “buried” is used to identify not outcropping faults.
9. Fault System Name (short name: F_S): name of the fault system consisting of a number of along-strike or perpendicular to strike interconnected Host Faults.
10. Fault System Onset (F_S_Onset): the age of the oldest recorded displacement and/or of the oldest syn-tectonic deposits among the Host Faults attributed to the FS.
11. Fault System Seismogenic Activity (F_S_Seism): evidence (yes, no, undetermined) of seismogenic or potentially seismogenic activity from integrated geological and seismological data.

The Host Fault traces are shown in Fig. 1a.

## Data Statistical Properties

**Fig. 4:**
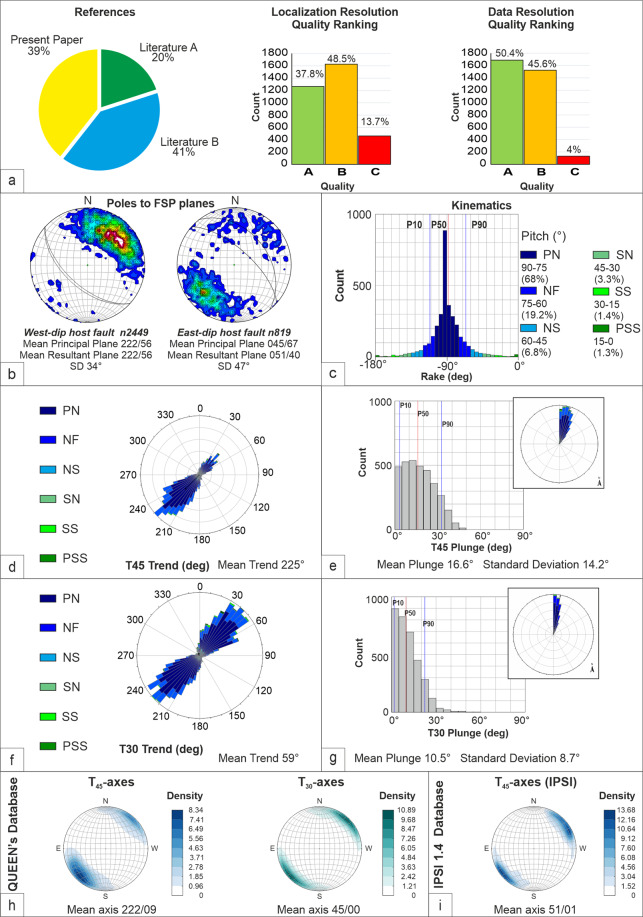
QUIN’s database statistical and quality characteristics. Key: FSP = Fault Striation Pair, T_45_ and T_30_ = extensional axes. PN = Pure Normal fault, NF = Normal Fault, NS = Normal Strike-slip fault, SN = Strike-slip Normal fault, SS = Strike-Slip fault, PSS = Pure Strike-Slip fault. (**a**) Cake diagram with percentage of FSP data first given in the present paper or derived from the literature (A-references containing one of the authors of this paper; B = exclusively other Authors); histograms representing quality rankings (A, B, C, where A is the best) for SS location and FSP data resolution. (**b**) Density contours of pole stereographic projections subdivided for Structural Sites (SS) lying on west- and east-dipping Host Faults (poles to planes). (**c**) Rake histogram with rake-based kinematic bins and corresponding pitches angles with relative frequency expressed in percent. (**d**) Trend rose diagram and plunge histogram (**e**) of the tensional deformation axis T_45_, calculated assuming the fracture angle θ = 45°. (**f**) Trend rose diagram and plunge histogram (**g**) of the tensional deformation axis T_30_, calculated assuming the fracture angle θ = 30°. (**h**) T-axis (T_30_ and T_45_) density contours from this database, compared with the (**i**) T-axis density contours of upper crust (depth < 15 km) focal mechanisms in the IPSI’s database^2^ within the boundaries of the QUIN study area.

We show here the statistical properties of the QUIN 1.0 data (Fig. 4). The 3339 FSP data are for ~60% derived from the literature and for ~40% from new observations, all ranked with quality parameters (A, B, C; A is best). When considering the Location Quality Ranking, 37.8% of the FSP data fall in class A, 48.5% in class B and 13.7% in class C. When considering the Data resolution quality ranking, 50.4% of the data fall in class A, 45.6% in class B and 4% in class C (Fig. 4a).

The FSP number within each SS varies from a minimum of 1 to a maximum of 46 data. ~50% of the SS have more than 6 FSP. SS with only one or very few FSP data, generally referred to as the major Host Fault plane, although not suitable for forthcoming stress inversion analysis, gives precious kinematic information on less studied or surveyed fault segments.

In Fig. 4b, we subdivided the overall dataset in SS located on west- and east-dipping Host Faults (76% and 24%, respectively) and represented the fault plane attitudes as density contour of the pole distributions. The corresponding best-fit planes dip 56° toward N222° and 52° toward N40, respectively. The FSP data associated with the east-dipping Host Faults (25%) show several mesoscopic antithetic faults (Fig. 4b), whereas the antithetic structures are minimal in association with SS sites located on the west-dipping Host Faults (75%). This difference may be attributed to the different outcropping conditions of the west- and the east-dipping faults. The former tend to occur associated with carbonate rocks and are characterized by a well-defined and easily recognizable main fault plane (Fig. 2). The latter may have a less discrete surface expression, especially where they occur associated with flysch deposits.

In Fig. 4c, the FSP fault kinematics is represented as a histogram (5° bin) of rake angle values. P10, P50 and P90 correspond to rake values of −112°, −88°, −66°, respectively, all falling within the normal fault type kinematics (pitch <60°). The prevailing dip-slip kinematics shown in the rake distribution is not surprising given the uniformity of the intra-Apennine Quaternary tectonic setting^18^ and is indicative of the scale invariance of the faulting process outlined by Allmendinger *et al*.^53^. A small percentage of data (<10%) shows strike-slip kinematics (pitch <30°), but the corresponding tensional axes remain coaxial with extensional deformation in an average SW-NE direction.

The rose diagrams and histograms in Fig. 4d,e represent the attitudes (trend and plunge) calculated for the FSP tensional axes referred to as T (θ = 45°) and T30 (θ = 30°), allowing a preliminary comparison between them.

Whereas T_45_ and T_30_ are almost coaxial in strike (common mode in direction N45), they significantly differ in plunge (Fig. 4e,g). Both T_45_ and T_30_ plunge distribution are unimodal but with a substantial difference. T-axes plunge shows the mode at an angle of 20°, with 50% of the data having a dip >20°. T_30_ has the mode at an angle of 10°, with 85% of the data having a dip <20°. It appears evident that when assuming the Andersonian conditions, which are reasonable near the surface^15^, the principal deformation axes T_30_-B_30_-P_30_, calculated assuming θ = 30°, assume a near-optimal orientation for most of the FSPs. The least principal strain directions (P) are largely near-vertical and the other two (B and T) near-horizontal. About 75% of the FSP dataset shows a T_30_ plunge lower than 20°, versus a value of 50% for T_45_-axes (Fig. 4d–g).

## Technical Validation

As mentioned, the QUIN’s database represents a collection of published and unpublished data on FSP extracted from tectonic maps, stereographic projections and tabulated data available since the early 1990s. With the lack of tabulated coordinates (*i.e*., Lat and Long) and fault/slip parameters, the precision in both the SS location and the FSP attitude depends on the degree of resolution of the original information (maps and stereographic projection). For these reasons, the precision is difficult to validate by any designed experiment. Nevertheless, we validated QUIN’s data considering two different quality rankings based on the resolution of the source information in terms of the precision of the SS location (Q_loc) and the reproducibility of the fault/slip data (Q_res; see Fig. 4a and Data Record section).

Nearly 60% of the FSP were surveyed by at least one of the authors of this paper (Present Paper and Literature A in Fig. 4a). This implies a general guarantee of homogeneity in FSP survey methodological criteria.

In databases like the one presented, there are sources of both aleatory and epistemic uncertainty, considered respectively as irreducible, inherent, and due to chance, and reducible, subjective, and due to lack of knowledge^104^. Being able to establish and quantify these uncertainties is a challenge as the data sources are manifold. The first source of aleatoric uncertainties arises from fieldwork, and it regards the precision in measuring fault planes and slickenlines. For newly and recently acquired data (using tablet computers), we performed systematic checks on attitudes using Brunton compasses and the GPS position. Other sources of uncertainties derive from the stages of data acquisition from maps and stereoplots of the literature, which could be a source of operator biases^105,106^. Furthermore, in the case of data reading from stereoplots, the striation may not lie precisely on the fault plane, thus making a correction needed. This type of correction has resulted in being always less than 2° in our database (7 of 3339 > 2°).

We highlight that the FSP rake shows a gaussian peak at −90°, with 606 data (about 18% of overall in the database) showing pure dip-slip kinematics (Fig. 4c). This peak is mainly due to data indicated as “dip-slip” in some of the geologists’ old booklets, meant as near pure normal faults. We evaluate that this may imply a rake error in the order of ±5°.

FSP data with strike-slip kinematics are present in a reduced percentage and are randomly distributed. The near sub-horizontal extensional-axis conditions (plunge ≤20°) are achieved by nearly 88% of the T_30_-axes corresponding to FSP with fault plane dip-angle ≥40°, and by nearly 63% of the T_45_-axes corresponding to FSP with fault plane dip-angle ≥25°. In general, the lower plunge values between T_45_- and T_30_-axes are observed for the T_30_-axes for fault dip-angle values ≥53° (~72% of FSP), and for T_45_-axes for fault dip-angle values <53° (~28% of FSP).

We recall that a common selection criterion for data to be inverted or represented as a projection on the horizontal plane is just having a plunge ≤20°, independently from the dynamic or kinematic meaning of the P- and T-axes.

No changes are observed for the neutral axis, which contains the fault direction in both cases.

Validation of the QUIN Quaternary database as representative of the Present deformation field can arise from comparing the QUIN’s deformation data with the P-B-T ones derived from the upper-crust focal mechanisms of the IPSI database^2^. The co-axiality between the long-term (*e.g*., Quaternary in this case) and the present-day instantaneous (seismological) deformation field is evident in the stereographic projections of Fig. 4h,i and is even more evident when considering the Andersonian-type strain axes (T_30_-B_30_-P_30_).

## Usage Notes

Possible uses of the QUIN database and supporting Host Fault database are to:

1. verify whether the present configuration of the intra-Apennine Quaternary fault system is developed in the course of a unique regional extensional phase or, instead, it is due to the alternation of non-coaxial extensional phases or extensional and strike-slip phases;
2. verify whether the long-term intra-Apennine Quaternary deformation field is coaxial or not, at the regional and local scales, with the present state of stress derived from focal mechanisms and geodetic data. In case of co-axiality, preliminarily observed comparing the stereographic projections in Fig. 4h,i, the QUIN database will be fundamental to filling spatial information gaps on the crustal state of stress left from instrumental earthquake data;
3. integrate the long-term outcropping deformation pattern derived from QUIN with the present deformation pattern at depth as derived from focal mechanisms to build multi-depth maps of horizontal Stress Trajectories (S_H_ min and max) and tensorial 3-D stress grids;
4. refine and locally extend the surface boundary of the Italian extensional seismogenic province;
5. make available a regional compilation of detailed fault traces and structural data to constrain the geometry and kinematics of the intra-Apennine potentially seismogenic faults, also performing detailed studies on kinematic partitioning and rupture segmentation, both with classical methodologies and with new technologies^11,107–109^;
6. interpolate the rake angle values among neighboring SS along the Host Fault strike and build gridded rake distributions for fault alignments to be used, for example, as one of the data inputs for fault-based SHA evaluations;
7. represent each SS having a sufficient and kinematically homogeneous fault population as pseudo-focal mechanisms to be compared with seismological focal mechanisms^10,108^;
8. perform formal fault stress inversion, with different approaches and algorithms, and utilize the regional stress information and the Host Fault attitude to calculate fault-slip tendency^36,110^, fault instability and Coulomb failures scenarios^111^;
9. once available, the QUIN database can be used for all the other applications identified by the World Stress Map compilers and may bring information on more local stress perturbation which may also be relevant to control fault activation during a multi-source event^10,77,112,113^.

Although generally underused in the active stress database^1,2^, geology-derived fault-slip data may represent a long-term stress regime still operating in an active region, such as Italy. Databases such as QUIN may be helpful for seismotectonic and seismic hazard investigation purposes, especially in areas that have not yet released earthquakes since historical times. In the following months, the database will be extended to southern peninsular Italy, with data recovered from the literature ad newly acquired within the frame of the MUSE-4D seismotectonic project, an Italian national project financed to some of us.

## Data Availability

The MATLAB code developed for this work is available from ZENODO^48^, together with a complete simple guide that illustrates fundamental steps to perform the kinematic analysis and graphical representation.
